# A serine/threonine phosphatase encoded by *MG_207* of *Mycoplasma genitalium* is critical for its virulence

**DOI:** 10.1186/1471-2180-13-44

**Published:** 2013-02-21

**Authors:** Mario A Martinez, Kishore Das, Sankaralingam Saikolappan, Luis A Materon, Subramanian Dhandayuthapani

**Affiliations:** 1 Regional Academic Health Center and Department of Microbiology and Immunology, University of Texas Health Science Center at San Antonio, Edinburg, TX, 78541, USA; 2 Department of Biology, University of Texas Pan American, Edinburg, TX, 78539, USA

**Keywords:** Serine, Threonine kinase, Phosphatase, Mycoplasma, Virulence, Pathogenesis

## Abstract

**Background:**

Bacterial signal transduction systems like two component system (TCS) and Serine/Threonine kinase (STK) and Serine/Threonine phosphatase (STP) play important roles in the virulence and pathogenesis of bacterial pathogens. *Mycoplasma genitalium*, a mollicute that causes the urogenital diseases urethritis and cervicitis in men and women, respectively, is a pathogen which lacks TCS but possesses STK/STP. In this study, we investigated the biochemical and virulence properties of an STP protein encoded by the gene *MG_207* of this species.

**Results:**

We overexpressed MG207 in *Escherichia coli* overexpression system as a recombinant His_10_MG207 protein and purified it with affinity chromatography. This recombinant protein readily hydrolyzed the substrate *p*-nitrophenyl phosphate (pNPP) in a dose-dependent manner. Additional studies using synthetic peptides as substrates revealed that the recombinant protein was able to hydrolyze the threonine phosphate. Further, a transposon insertion mutant strain of *M. genitalium* (TIM207) that lacks the protein MG207 showed differentially phosphorylated proteins when compared to the wild type G37 strain. Mass spectrometry revealed that some of the key proteins differentially phosphorylated in TIM207 strain were putative cytoskeletal protein encoded by the gene *MG_328* and pyruvate dehydrogenase E1 α chain encoded by the gene *MG_274.* In addition, TIM207 was noticed to be less cytotoxic to HeLa cells and this correlated with the production of less hydrogen peroxide by this strain. This strain was also less efficient in inducing the differentiation of THP-1 cell line as compared to wild type *M. genitalium*.

**Conclusions:**

The results of the study suggest that MG207 is an important signaling protein of *M. genitalium* and its presence may be crucial for the virulence of this species.

## Background

Bacteria adapt to changing environments by regulating their gene expression through signal transduction systems. Two kinds of signal transduction systems exist in bacteria; the two component system (TCS) and serine/threonine kinases (STK) and phosphatases (STP) system [1-4]. Although both systems transduce signals by phosphorylation events, they have distinct ways of doing this. While TCS uses a sensor histidine kinase and a regulator protein to transduce the signals, the STK /STP regulate gene expression by protein-protein interaction [3,4]. However, it should be noted that not all kinases and phosphatases associated with serine or threonine residues in prokaryotes are STK/STP. The STK/STP has special signature motifs [5,6] and is restricted to selected species of bacteria. It was once thought that bacteria have only TCS but not STK/STP. However, evidence for the occurrence of STK/STP in bacteria continues to accumulate [4]. Also, it has been reported that bacterial TCS and STK/STP systems cross talk with each other [7].

In addition to their role in the physiology, STK/STP plays a significant role in the virulence of some pathogenic bacteria, including bacteria relevant to public health such as Yersinia and Mycobacteria [4,8]. For instance, YpkA, an STK of *Yersinia pseudotuberculosis*, is critical for the disruption of host cytoskeleton during infection [9,10]. In *Mycobacterium tuberculosis*, lack of STK PknG and PknH has been reported to show reduced viability of this bacterium and increased bacterial load, respectively, in mouse models [11,12]. The significance of STK in the pathogenesis of *Staphylococcus aureus*[13,14], *Streptococcus pneumoniae*[15], *S. pyogenes*[16], *Pseudomonas aeruginosa*[17], *S. agalactiae*[18,19], *Mycoplasma pneumoniae*[20] and *Salmonella enterica*[21] has also been reported. With respect to STP, relatively few studies have been undertaken in understanding their role in bacterial virulence and most of them focus on Pneumococcus [4]. An STP (SP-STP) of *S. pyogenes* is required for the production of hemolysin and to cause apoptosis in the host cells [16,22,23]. Its homologue, STP1, in group B *Streptococcus sp* is also associated with the production of hemolysin and lack of this STP leads to less efficient systemic infection by this bacterium [24]. Very recently, an STP (PhpP) of *S. pneumoniae* is found to have a role in the adherence of this species [25]. Besides, an STP of *Listeria monocytogenes* is reported to be essential for the growth of this bacterium in murine model [26].

*Mycoplasma genitalium* is a bacterium that lacks a cell wall and is one of the smallest self-replicating organisms with a genome size of 580 kb [27]. It is the etiological agent for the diseases non-gonococcal urethritis and cervicitis in men and women, respectively [28,29]. In women, it is also implicated in diseases like endometritis, pelvic inflammatory syndrome and tubal infertility [30-32]. Additionally, *M. genitailum* coinfection in HIV patients has been reported to have increased shedding of HIV in urogenital mucosal regions of the female [33]. Although it was initially thought that *M. genitalium* primarily attaches with epithelial cells of the host to cause the disease, evidences indicate that it invades epithelial cells and is localized on the periphery of the nucleus of the infected cells [34,35]. The intracellular *M. genitailum* is reported to persist within the infected cells for a long time [34,36]. It should be noted that intracellular survival and persistence of this bacterium may require signal transduction mediated adaptation, as do other bacteria in similar circumstances [37-39]. Strikingly, however, *M. genitalium* and its close relative *M. pneumoniae* are lacking the classical bacterial TCS [27,40,41], although a few mycoplasmas like *M. penetrans* and *M. iowae* do have TCS (NCBI data base). Besides, both species have only a limited number of regulators controlling gene expression at the transcription level [27,40], and this has been attributed to their small genomes due to reductive evolution. Nevertheless, these species have genes encoding STK and STP [27,40,41]. In fact, the STK of *M. pneumoniae* has been demonstrated to have an effect on the adherence of this species [20], although no such effect was noticed with an STP of this species (PrpC) [42].

Our long term objective is to determine the roles of STK and STP in *M. genitalium* pathogenesis and signal transduction. NCBI database of *M. genitalium* genome sequence [27] reveals that this bacterium possesses a gene encoding STK (*MG_109*) and three genes encoding STP (*MG_108*, *MG_207* and *MG_246*). We initiated our studies first with *MG_207* because we had a mutant strain for this gene readily available from a transposon mutant library [43]. Here, we show that MG207 is an alkaline phosphatase and it dephosphorylates threonine phosphate. We also report that *M. genitalium* lacking in MG207 (TIM207 strain) shows differentially phosphorylated proteins in two-dimensional gels. In addition, we provide evidence that TIM207 has reduced virulence as compared to wild type *M. genitalium*.

## Results and discussion

### *MG_207* encodes a functional phosphatase

**Figure 1:**
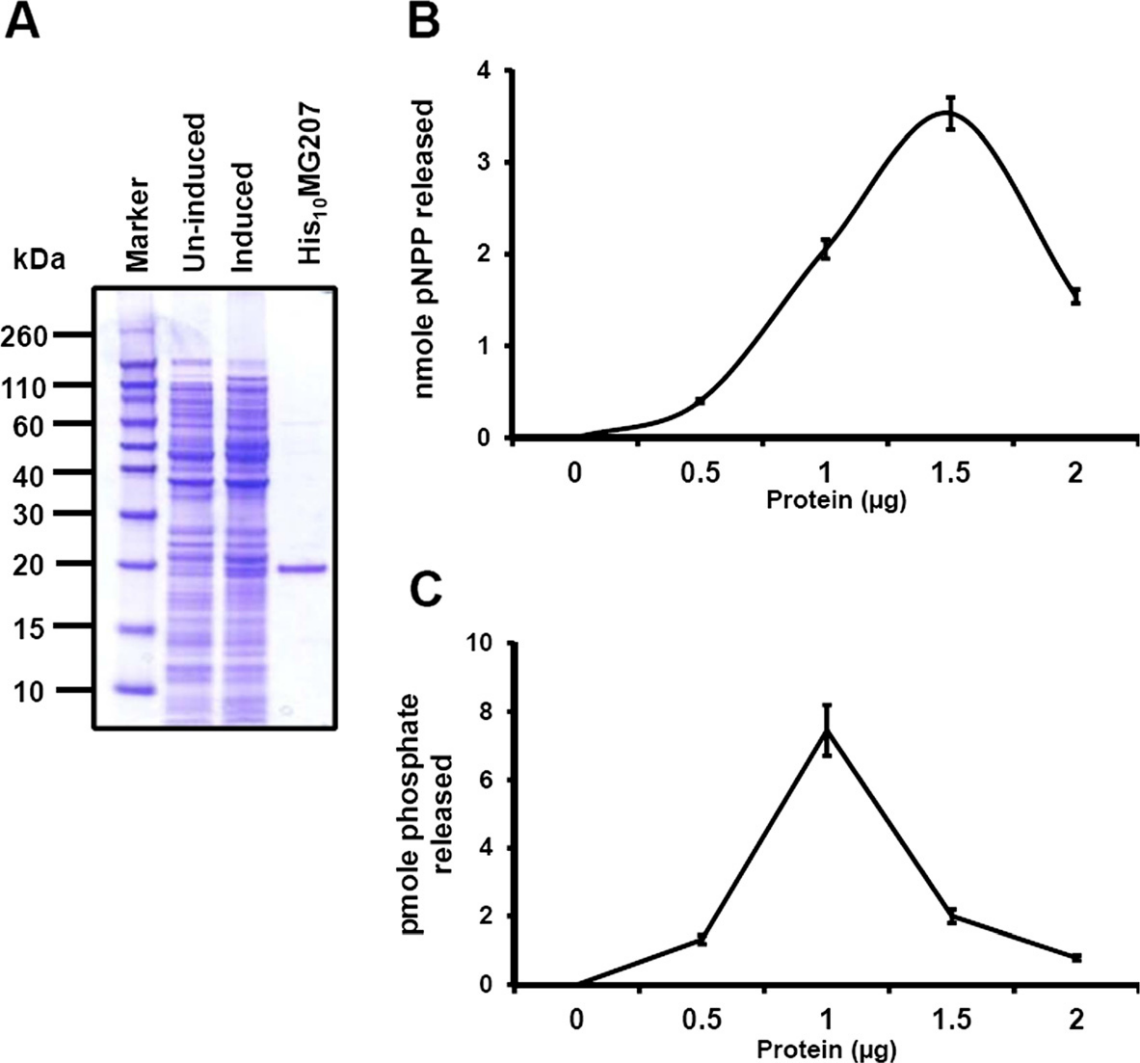
**Production of recombinant His**_**10**_**MG207 protein and determination of phosphatase activity. A**. Protein profiles of overexpressed and purified protein of His_10_MG207. Lanes: Marker, EZ-Run Rec Protein Ladder (Fisher Scientific); Un-induced, extracts of *E. coli* strain BL21 before the addition of 0.5 mM IPTG; Induced, extracts of *E. coli* strain BL21 after 3 h of the addition of 0.5 mM IPTG; His_10_MG207, purified His tagged MG207 protein after Ni-NTA affinity chromatography. Numbers on the left represent the sizes of the marker proteins in KDa. **B**. Phosphatase activity of His_10_MG207 with pNPP as substrate. Various amounts (μg) of purified His_10_MG207 protein (Protein) were added to the reaction mixture containing pNPP. Activity was measured in the presence of 5 mM MgCl_2_. Values represent Mean ± SD. **C**. Phosphatase activity of His_10_MG207 with synthetic threonine peptide as substrate. Various amounts (μg) of purified His_10_MG_207 protein (Protein) were added to the reaction mixture containing synthetic threonine phosphate (KRpTIRR). Values represent Mean ± SD.

The gene *MG_207* is predicted to encode a serine/threonine phosphatase. To verify this, we created the plasmid pMG207EX to overexpress MG207 protein in *E. coli.* This plasmid was transformed into *E. coli* BL21 (DE3) strain and induced with IPTG. Figure 1A shows the overexpression of His_10_MG207 protein by *E. coli* harboring the plasmid pMG207EX and its purification. The purified His_10_MG207 protein separated onto SDS-PAGE and stained with coomassie blue exhibited a size of 19 kDa (Figure 1A). This correlated with the predicted size of 18.5 kDa of *MG_207*.

To determine if the purified His_10_MG207 protein was functional, we assayed the phosphatase activity of this protein using the substrate *p*-nitrophenyl phosphate (pNPP). The His_10_MG207 readily hydrolyzed pNPP in a dose dependent manner (Figure 1B) in the alkaline pH of 8.0. To rule out the possibility that the observed phosphatase activity of His_10_MG207 was not due to *E. coli* host derived phosphatase, we used similarly overexpressed and purified His_10_Ohr protein of *M. genitalium* as a control. Reactions with this protein (His_10_Ohr) or reactions with heat inactivated His_10_MG207 or reactions with His_10_MG207 but without Mg^2+^ in the reaction mixture showed no color formation with pNPP (data not shown), indicating that the overexpressed protein was a functional phosphatase dependent upon Mg^2+^ ion for its activity. To further assess whether His_10_MG207 is a serine/threonine phosphatase, malachite green based phosphatase assay was performed using synthetic serine (RRApSSVA) or threonine (KRpTIRR) phosphopeptide. No activity was noticed with either peptide in the presence of Ni^2+^, a cation supplied with the assay kit (data not shown). However, substitution of Ni^2+^ with Mg^2+^ in the reaction mixture released the phosphate from threonine peptide (Figure 1C), but this failed to release the phosphate from serine peptide. We presume that the absence of activity with the serine phosphate peptide may be due to the requirement of appropriate conditions. Alternatively, it is possible that the serine phosphate in this particular peptide is un-accessible for the enzyme. However, the fact that MG207 requires a metal (Mg^2+^) for its activity with pNPP or with threonine peptide suggests that it is a metal dependent phosphatase.

This observation is consistent with reports of other STPs like Stp of *L. monocytogenes*[26], PhpP of *S. pneumoniae*[44], PrpC of *M. pneumoniae*[42] and Stp1 of *S. agalactiae*[18], all of which required divalent metal cofactor Mn^2+^ for their activity. In bacteria, STP belongs to two families, phosphoprotein phosphatases (PPP) and metal dependent phosphatases (PPM). The major difference between these two groups appears to be their specificity for substrates. While PPM specifically hydrolyzes serine or threonine phosphates, the PPP hydrolyzes, in addition to serine and threonine phosphates, histidine and tyrosine phosphates [45]. Although PP2C phosphatase, a member of the PPM family, has some catalytic similarities with PPP, this does not show any amino acid similarity with PPP [46]. Further, it appears that MG207 is only a closely related protein to PP2C phosphatase, because the cluster of orthologous groups (COGs) classification has placed this protein in a different group of bacterial phosphatase.

### TIM207 strain and its confirmation

**Figure 2:**
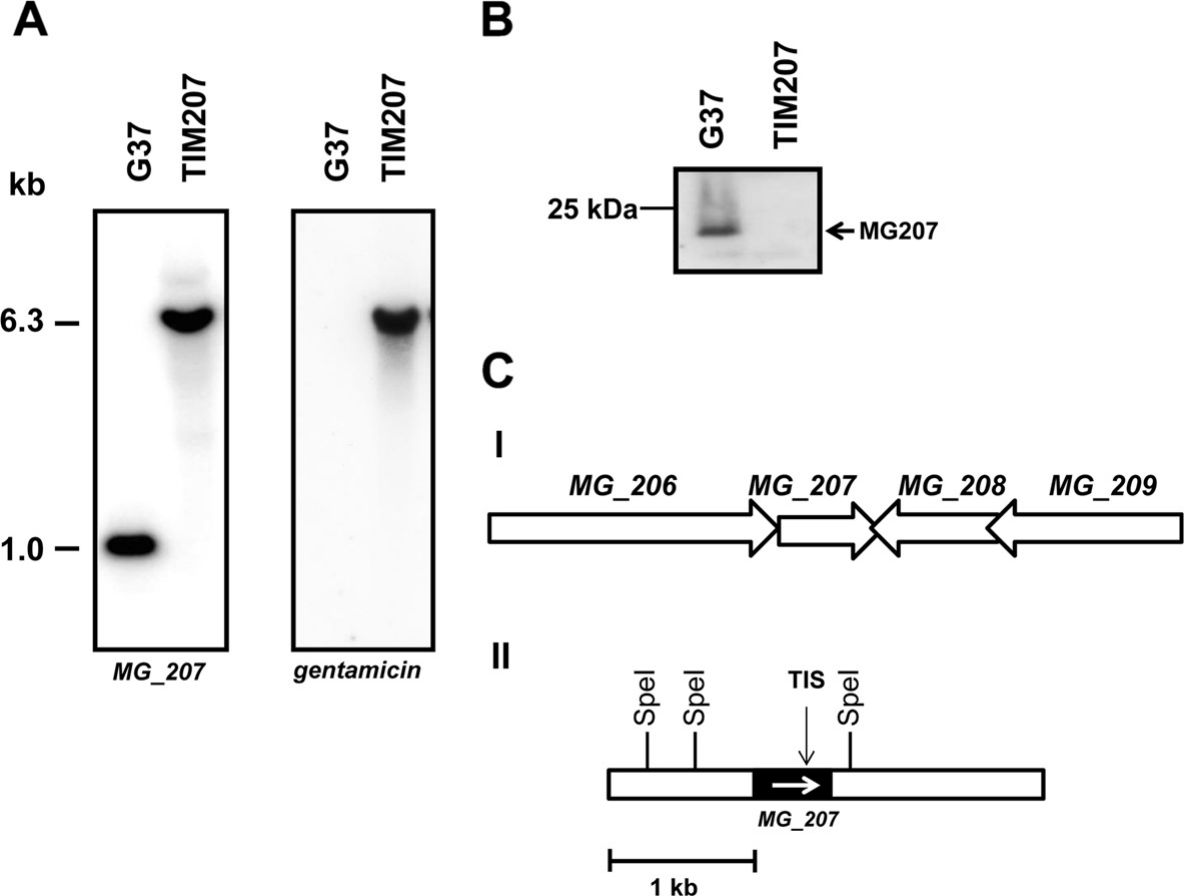
**Confirmation of gene disruption in *MG_207 *by Southern and immunoblot analyses. A**. Southern analysis of *M. genitalium* DNA from wild type G37 and TIM207 *strains.* Membranes were probed with radiolabeled *MG_207* and *gentamicin* gene sequences. G37 and TIM207 represent *M. genitalium* wild type and *MG_207* mutant strains. Sizes of DNA fragments are indicated in kilo bases (kb). **B**. Immunoblot analysis of wild type G37 and TIM207 strains. SDS-PAGE separated proteins were transferred to nitrocellulose membrane and probed with anti-His_10_MG207 rabbit antiserum (1:500). After treating with peroxidase labeled second antibody (1:10,000 dilution), blots were developed with chemiluminiscent method (ECL) and the signals autoradiographed. G37 and TIM207 represent *M. genitalium* wild type and *MG_207* mutant strains, respectively. The size (kDa) of the marker protein is given on the left. **C**. Schematics showing the organization of *MG_207* in the genome of *M. genitalium*. I. Organization of genes around *MG_207*. Arrows represent genes and their direction of transcriptions. Numbers above the arrows indicate the assigned number of each gene. II. Restriction sites around *MG_207* gene. Open boxes represent regions adjacent to *MG_207*: Black box represents the gene *MG_207*. Arrow within the black box indicates the direction of transcription of *MG_207*. SpeI indicates the locations of SpeI restriction site around *MG_207*. TIS indicates the site of transposon insertion.

To understand the role of MG207 in signal transduction and pathogenesis of *M. genitalium*, we sought to create a mutant strain through homologous recombination. However, we were able to acquire a similar mutant strain from *M. genitalium* Tn4001 transposon mutant library generated by Dr. John Glass [43]. The insertion of Tn4001 in the coding region of *MG_207* had already been determined by sequencing [43]. In order to reconfirm this insertion and to check if this strain has any additional Tn4001 insertions due to sub-culturing, we probed the genomic DNA of *M. genitalium* wild type G37 strain and TIM207 cut with SpeI, in Southern hybridization. The membrane hybridized with radiolabeled DNA of *MG_207* revealed strong signals around 1.0 kb in the G37 strain and 6.3 kb in the TIM207*.* In addition, a weak signal was also noticed in the TIM207 strain around 8.0 kb region (Figure 2A). The shift in hybridization signals for *MG_207* and also the presence of additional signals for *MG_207* in TIM207 strain, as compared to G37 strain, reconfirmed that the gene was disrupted by Tn4001 insertion. In addition, hybridization of the blot with gene fragment coding for gentamicin resistance, which is part of the transposon, revealed signals only at one position which corresponded to the signals of *MG_207* in the TIM207 strain (Figure 2A). This indicated that this strain has no additional Tn4100 insertions in the chromosome and the mutant is stable.

Further, to determine whether the transposon insertion indeed disrupted the expression of MG207 protein, we analyzed the proteins of G37 and TIM207 strain in immunoblot with anti-MG207 antiserum. This antiserum detected the MG207 protein only in the wild type G37 strain and not in the TIM207 strain (Figure 2B), indicating that the disruption of the gene affected the expression of the protein. We do not expect that Tn4001 insertion in this strain (TIM207) will have any polar effects on its downstream genes, because the transcription of the downstream genes is predicted to be in the opposite orientation (Figure 2C). This situation implies that complementation of the TIM207 with a functional allele to assess the function of MG207 is of limited significance. Moreover, the only way by which the *M. genitalium* mutant strain can be complemented is through the use of a transposon which can insert a copy of the functional allele of the mutated gene in an unknown location of the chromosome. It is very likely that the unknown location may be a functional gene and this will affect the interpretation of the complimented phenotype. Therefore, we have used a *M. genitalium* strain called TIM262, which bears the same transposon as in TIM207, inserted in the gene *MG_262*, as a control strain in some experiments. The gene *MG_262* is predicted to code for a 3´-5´ exonuclease (NCBI database).

### TIM207 strain exhibits differentially phosphorylated proteins

**Figure 3:**
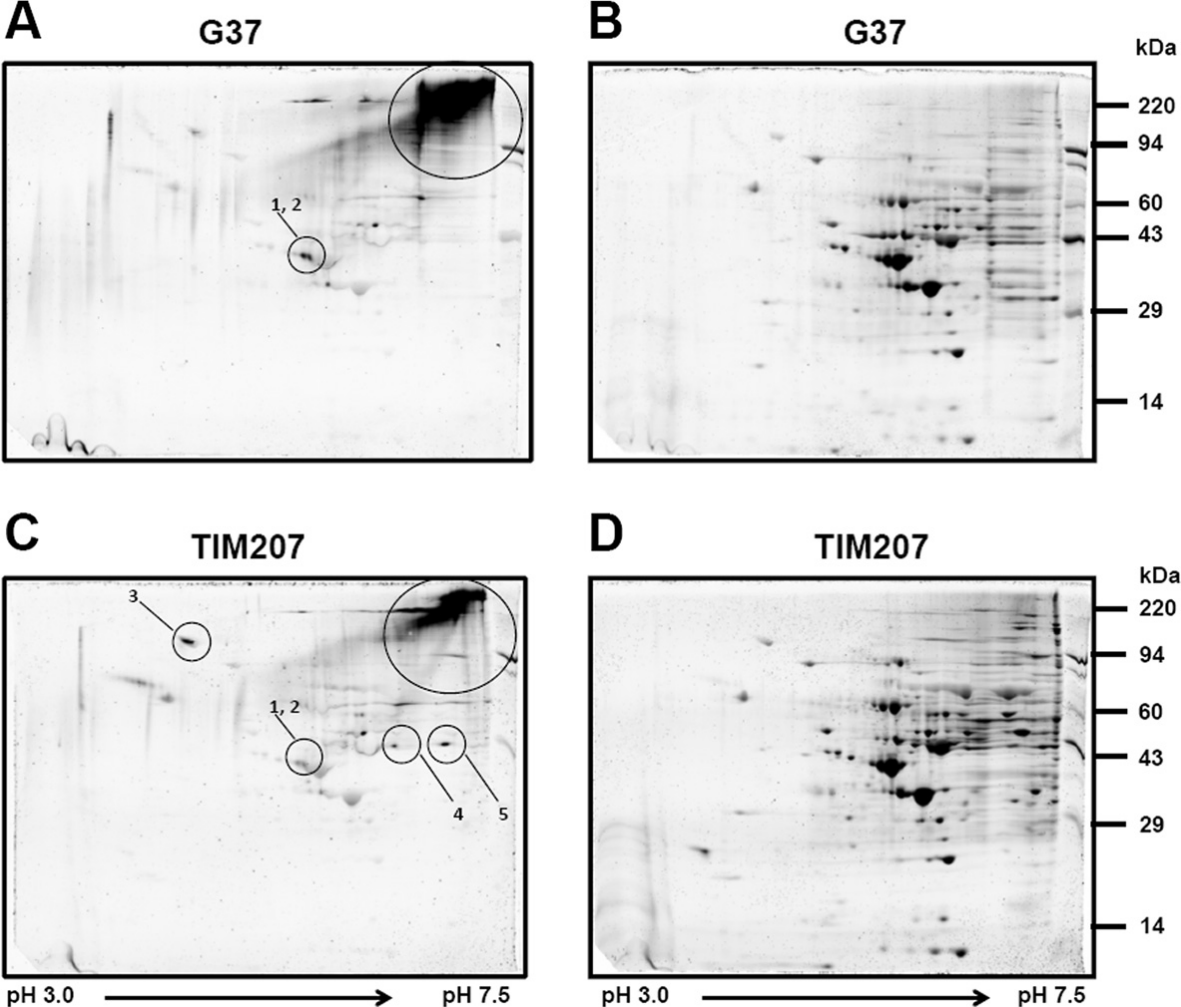
**2D gel analysis of *M. genitalium *total and phosphorylated proteins.** Total protein from *M. genitalium* strains (G37 wild type and TIM207 mutant) were separated in 2D gels and stained with Pro-Q Diamond and Sypro Ruby for the detection of phosphoproteins (gels **A** and **C**) and total proteins (Gels **B** and **D**), respectively. Protein spots circled and numbered are the ones subjected to mass spectrometry analysis. Protein spots shown in large circles denote the putative high molecular weight proteins showing differential phosphorylation. The sizes (kDa) of protein markers are shown on the right and direction of the first runs are shown by arrows.

As MG207 is a phosphatase presumed to be associated with signaling, it was predicted that absence of this protein might alter the phosphorylation status of some *M. genitalium* proteins. To determine this, and also to identify some of the differentially phosphorylated proteins, we performed 2-D gel analysis of proteins from G37 and TIM207 strains and stained them with Pro-Q Diamond (Figure 3A and C) and Sypro Ruby stains (Figure 3B and D). While the total proteins stained with Sypro Ruby showed similar profiles for G37 and TIM207 strains, the phosphoproteins stained with Pro-Q Diamond displayed different profiles for these strains. These differences in phosphorylation appear not due to differences in the growth of the wild type (G37) and mutant (TIM207) strains as they showed no significant differences (data not shown) in growth. Further, the differences do not appear due to variability in viability because both strains exhibited similar viability at the time of harvest (Additional file 1: Figure S1).

The predominant difference was noticed to be at the high molecular weight (HMW) areas which are shown in large circles (Figure 3A and C). As can be seen, the gels from G37 showed relatively dense and larger stained areas as compared to gels from the TIM207 strain, suggesting that some HMW proteins are less phosphorylated in TIM207 strain. However, these dense areas have shown no corresponding protein spots in Sypro Ruby stained gels, thus indicating that these areas do not represent real proteins but represent some artifacts. Therefore, we focused only on well separated and differentially phosphorylated proteins. These included two proteins (shown in circles 1 and 2) which showed relatively dense staining in the gels of G37 strain but were weaker in the gels of TIM207 strain, and three proteins (shown in circles 3, 4 and 5) that showed stronger staining in the gels of TIM207 strains but were weaker in the gels of G37.

To identify the differentially phosphorylated proteins, we subjected the protein spots 1–5 to mass spectrometry (Additional file 2: Table S1). Based on the number of peptide hits for each spots, the spots were identified as four different proteins (Table 1) namely, pyruvate dehydrogenase complex E1 subunit α (MG274), a putative cytoskeleton protein (MG328), a conserved hypothetical protein (MG281) and thymidine phosphorylase (MG051). Interestingly, our study validates the findings of a previous study which identified pyruvate dehydrogenase E1 α chain and the protein encoded by *MG_328* as phosphoproteins of *M. genitalium*[47]. Recently, both these proteins were found on the surface of *M. genitalium*[48], thus suggesting the possibility that they can play a role in *M. genitalium*-host interaction. What is intriguing in this study, however, is the reduced phosphorylation of a protein (PDH) in a phosphatase (MG208) deficient mutant of *M. genitalium*. Theoretically, if proteins are dephosphorylated by a specific phosphatase, then the expectation, in the absence of the phosphatase, is no change in the phosphorylation levels or increased levels of phosphorylation of proteins. In fact, the proteins identified in *PrpC* mutant of *M. pneumoniae*[49] behave in this manner. The differentially phosphorylated proteins identified in this mutant included RopE, an adhesion related protein P41, HMW3, MPN256 and MPN474. These proteins showed phosphorylation only in the *PrpC* mutant but not in the wild type. The contrasting situation in *MG_207* tends to speculate that additional protein(s) with possible kinase property is affected in this mutant and this putative protein fails to phosphorylate some proteins in the mutant. However, this is only a hypothesis and requires additional studies for confirmation. Nevertheless, differential phosphorylation of proteins has been shown in the STP mutant strains of *L. monocytogenes* and *S. pneumoniae*[44]. In addition to changes in protein phosphorylation, an STP (stp1) mutant of Group B *Streptococcus* has been shown to have altered expression of 294 genes which included *stk1*, the gene encoding a major kinase [24].

**Table 1:** Phosphorylated proteins identified by Mass spectrometry

**Spot number**^**a**^	**Protein name**	**Gene symbol**	**Gene**	**Putative function**
1	Pyruvate dehydrogenase E1 subunit α	*pdhA*	*MG_274*	Metabolism/surface protein
2	Pyruvate dehydrogenase E1 subunit α	*pdhA*	*MG_274*	Metabolism/surface protein
3	Putative cytoskeletal protein	*-*	*MG_328*	Cytoskeletal involvement
4	Conserved hypothetical protein	*-*	*MG_281*	Unknown
5	Thymidine phosphorylase	*deoA*	*MG_051*	Metabolism

^a^ Refers protein spots marked in Figure 3A and C.

### Ability of TIM207 strain to adhere and invade eukaryotic cells

**Figure 4:**
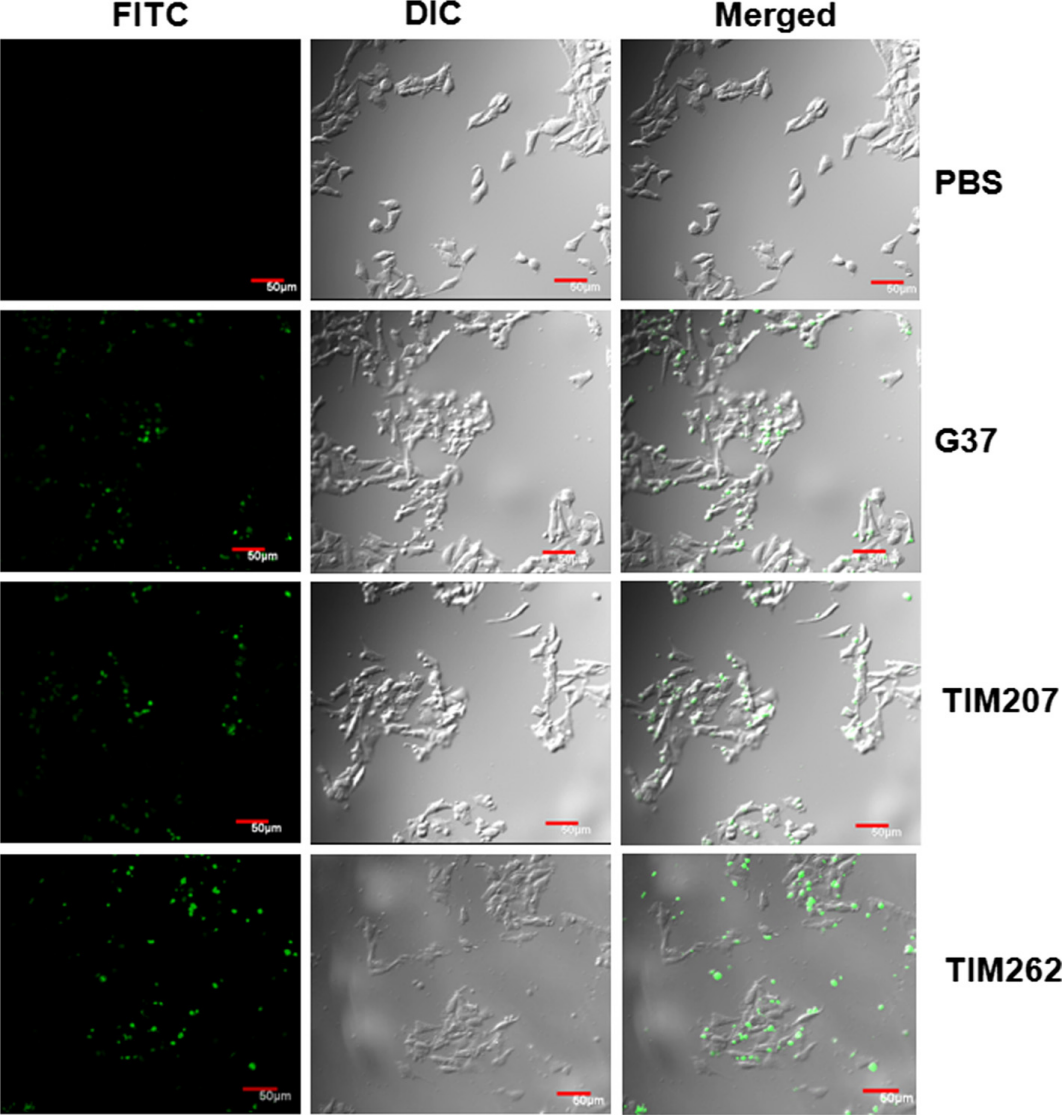
**Microscopic observation of adherence/invasion of *M. genitalium *strains to HeLa cells: FITC labeled *M. genitalium *strains were used to infect HeLa cells with MOI of 1:25 for 1 h as described in material and methods and observed with confocal microscopy.** G37, TIM207 and TIM262 indicate infection of cells with *M. genitalium* wild type G37 strain, *MG_207* mutant strain and control strain TIM262, respectively. PBS indicates uninfected control.

Since TIM207 strain showed differential phosphorylation of proteins, we speculated that this would have some impact on the adherence of this strain with eukaryotic cells. Consistent with this notion, we noticed that TIM207 strain adhered only partially to culture flasks when grown in SP-4 medium. The non-adherent mycoplasmas were seen as floating cell suspensions and this phenotype was highly reproducible. However, growing of only the floating or adherent cells resulted into adherent and floating cells in subsequent cultures, indicating that the non-adherent phenotype to the culture flask is a transient phenomenon. Further, since surface attachment of *M. genitalium* to culture flasks often correlates with adherence to eukaryotic cells, we tested the TIM207 strain for hemadsorption with sheep erythrocytes, a technique that we routinely use to assess the adherence of this species, and compared its phenotype with G37. TIM207 showed a hemadsorption positive phenotype similar to that of wild type G37 (data not shown), suggesting that there is no difference between these two strains with regard to adherence to eukaryotic cells. To evaluate this further, the ability of TIM207 strain to adhere/invade epithelial cells was also assessed by infecting HeLa cells. Confocal microscopic analysis of the infected cells revealed that both G37 and TIM207 strains exhibit similar levels of adherence/invasion, although the control strain TIM262 was little different from these by showing relatively higher levels of adherence/invasion (Figure 4). The reason for this difference is unknown at present. However, the fact that both G37 and TIM207 show more or less similar phenotype reiterates that the partial non-adherence to culture flasks by TIM207 strain has no correlation with its adherence to or invasion of eukaryotic cells. It has been shown [35] that invasion of *M. genitalium* into cultured HeLa and EM42 cervical epithelial cell lines occurs within 30 minutes after postinfection and the invaded bacteria are localized within nuclei. Interestingly, this study has also reported that only a subset of the bacteria (*M. genitalium*) invades the cells. This fact was confirmed by another group that used electron microscopy and they estimated that the invading bacteria would be around 50% of the total bacteria showing adherence [50]. In this context, it will be of interest to know whether there exists any difference between the wild type and TIM207 in the quantity of invading bacteria and this question will be addressed in our future studies.

Nevertheless, the partial culture flask non-adherence phenotype that we observed with the TIM207 strain is different from that of the completely culture flask non-adhering phenotype of *M. genitalium* strain reported earlier [51]. Feldner et al. [52] reported that adherence of mycoplasma to culture flasks are based on electrostatic forces rather than adhesion mediated. It is likely, therefore, that change in phosphorylation of some surface proteins, due to the absence of MG207, leads to change in membrane potential which ultimately affects the electrostatic force. In the case of *M. pneumoniae*, it is the STK, but not STP (PrpC), mutant which failed to adhere with culture flasks [20,42]. Consistent with this negative adherence to culture flasks, this STK mutant strain (*MPN248* mutant) exhibits reduced levels of adherence related proteins, including P1, in SDS-PAGE. However, recent studies have demonstrated that deletion of STP in strains of *S. pyogenes* (M1SF370) [22] and *S. pneumoniae* (D39)[25] leads to reduced adherence to pharyngeal cells. It appears, therefore, that disruption of both STK and STP can lead to adherence negative phenotype but it varies from species to species. However, the mechanism behind partial adherence of TIM207 to cultures flask remains elusive and it requires further study.

### TIM207 strain is less cytotoxic to HeLa cells

**Figure 5:**
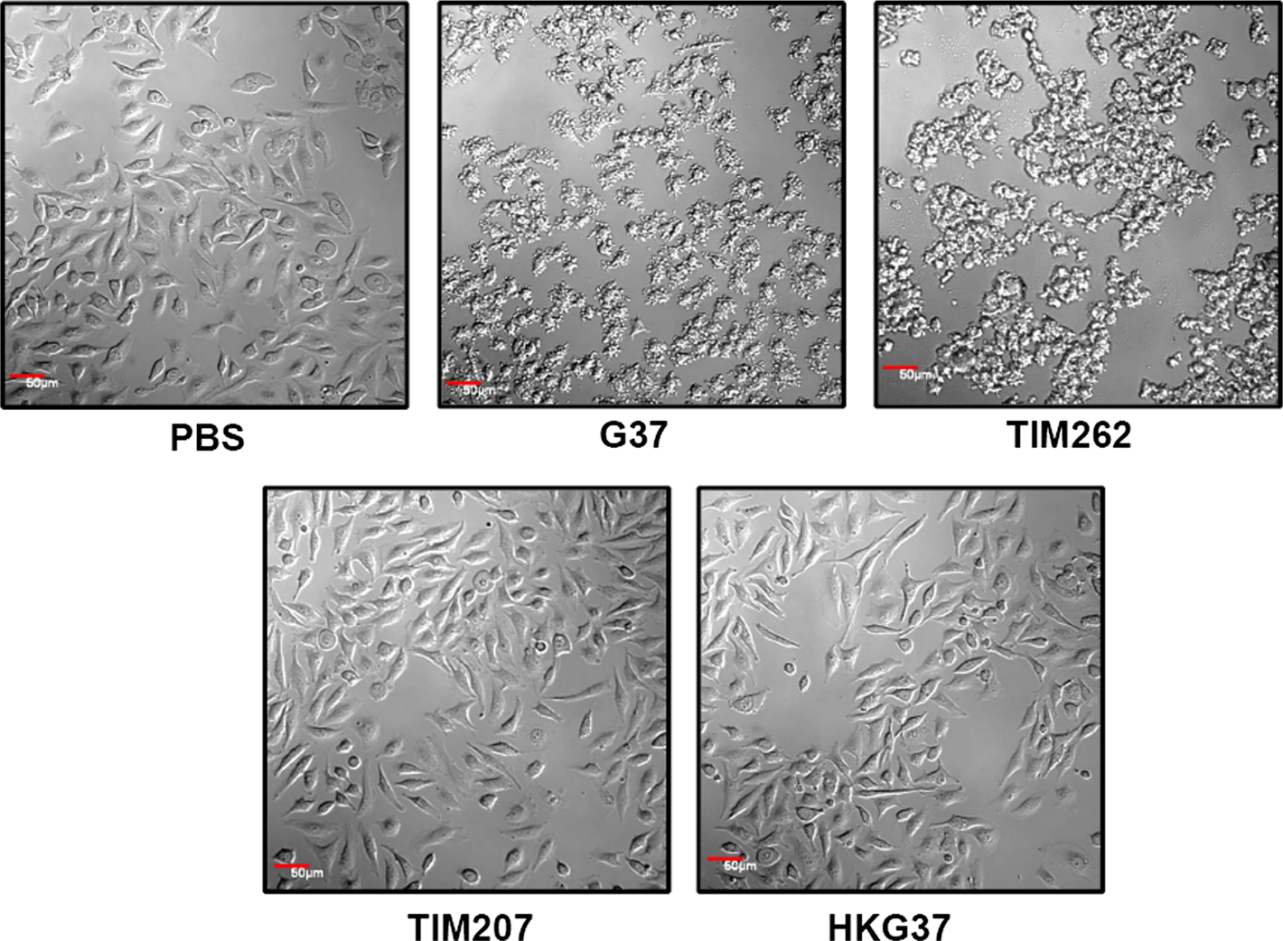
**Microscopic observation of cytotoxic effect by *M. genitalium *strains.** Cervical epithelial (HeLa) cells were plated in triplicate on microscopic slides and infected with *M. genitalium* G37 and TIM207 strains at an MOI of 1:50. Cells were observed with confocal laser microscope with 20X objective after 2–3 h incubation at 37°C. PBS indicates un-infected cells; G37, TIM207, TIM262 and HKG37 indicate infection of cells with *M. genitalium* wild type G37 strain, TIM207 mutant strain, TIM262 control strain and heat killed G37 bacteria, respectively.

**Figure 6:**
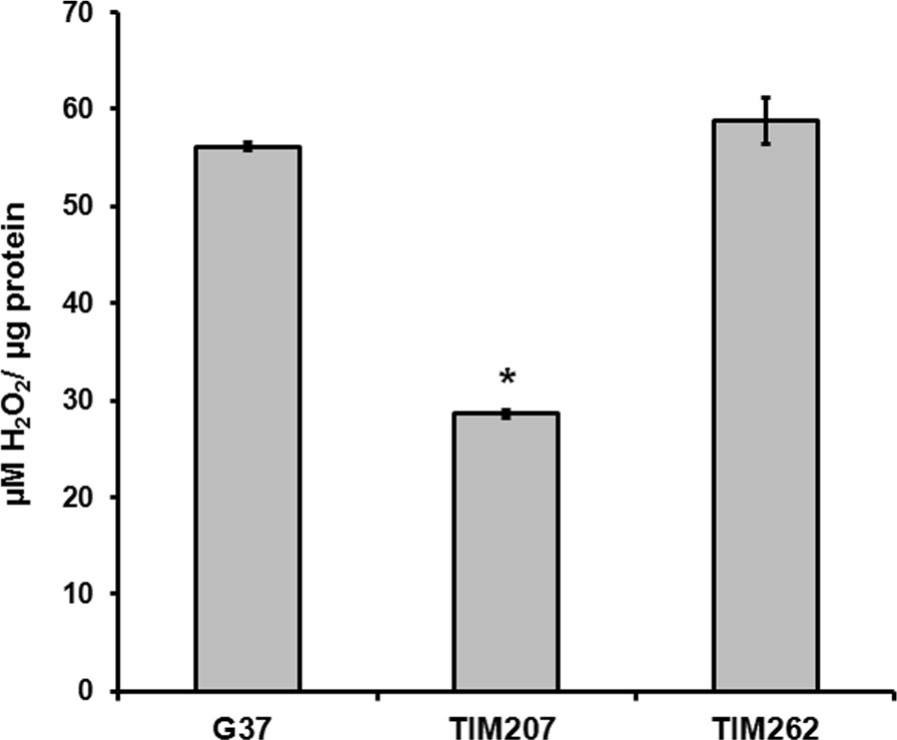
**Hydrogen peroxide (H**_**2**_**O**_**2**_**) production by *M. genitalium *cells.** Cells of *M. genitalium* strains (G37 wild type, TIM207 mutant and TIM262 mutant control) were sonicated in PBS and the presence of H_2_O_2_ in each sample was determined by FOX assay at 560 nm. The amount of hydrogen peroxide in each sample was determined using standard curve generated with H_2_O_2_ and the values expressed as μmoles H_2_O_2_/ μg protein. * indicate significant difference from G37 and TIM262 (p≤0.05).

Further to understand whether the lack of MG207 has any effect on other pathogenic mechanisms of *M. genitalium*, we examined the ability of TIM207 strain to cause cytotoxicity. Therefore, we infected HeLa cells with TIM207 and other control strains. Figure 5 shows the confocal microscopy observation of HeLa cells infected with *M. genitalium* strains. As can be seen, *M. genitalium* wild type strain G37 and a control strain TIM262*,* which hasTn4001 insertion in *MG_262* encoding 3´-5´ exonuclease*,* had severe cytotoxic effects on HeLa cells, while TIM207 had no such effect and behaved similar to that of heat killed G37 (HKG37) strain. Since cytotoxicity of mycoplasmas is due partly to the release of hydrogen peroxide by these species, we speculated that differences in cytotoxicity between the wild type and the mutant strains might be due to differences in the production of H_2_O_2_ by these strains. To rule out this possibility, we determined the H_2_O_2_ levels in these strains by FOX assay. The results showed significantly reduced levels of H_2_O_2_ in TIM207 strain as compared to G37 strain (Figure 6). This indicated that deletion of *MG_207* had some direct or indirect effect on the synthesis of H_2_O_2_ by *M. genitalium*. Mycoplasmas produce H_2_O_2_ by oxidizing the glycerophosphate of the glycolytic pathway by glycerophosphate oxidase [53]. It is likely that phosphorylation or dephosphorylation of some of the enzymes associated with this pathway leads to reduced production of H_2_O_2_ in TIM207 strain. Besides, in *M. pneumoniae* reduced cytotoxicity and H_2_O_2_ production is linked to reduced ability to utilize glycerol [20]. To understand if the reduced H_2_O_2_ production by TIM207 has any correlation with glycerol utilization, we determined the growth of the TIM207 strain in SP-4 medium containing glycerol instead of dextrose. Results presented in Additional file 3: Figure S2 reveal that this strain has a defect in the utilization of glycerol as compared to the wild type strain. These results, taken together, reiterate that reduced cytotoxicity of TIM207 is due partly to generation of relatively lower amount of H_2_O_2_ by this strain.

### TIM207 strain fails to differentiate THP-1 cells

**Figure 7:**
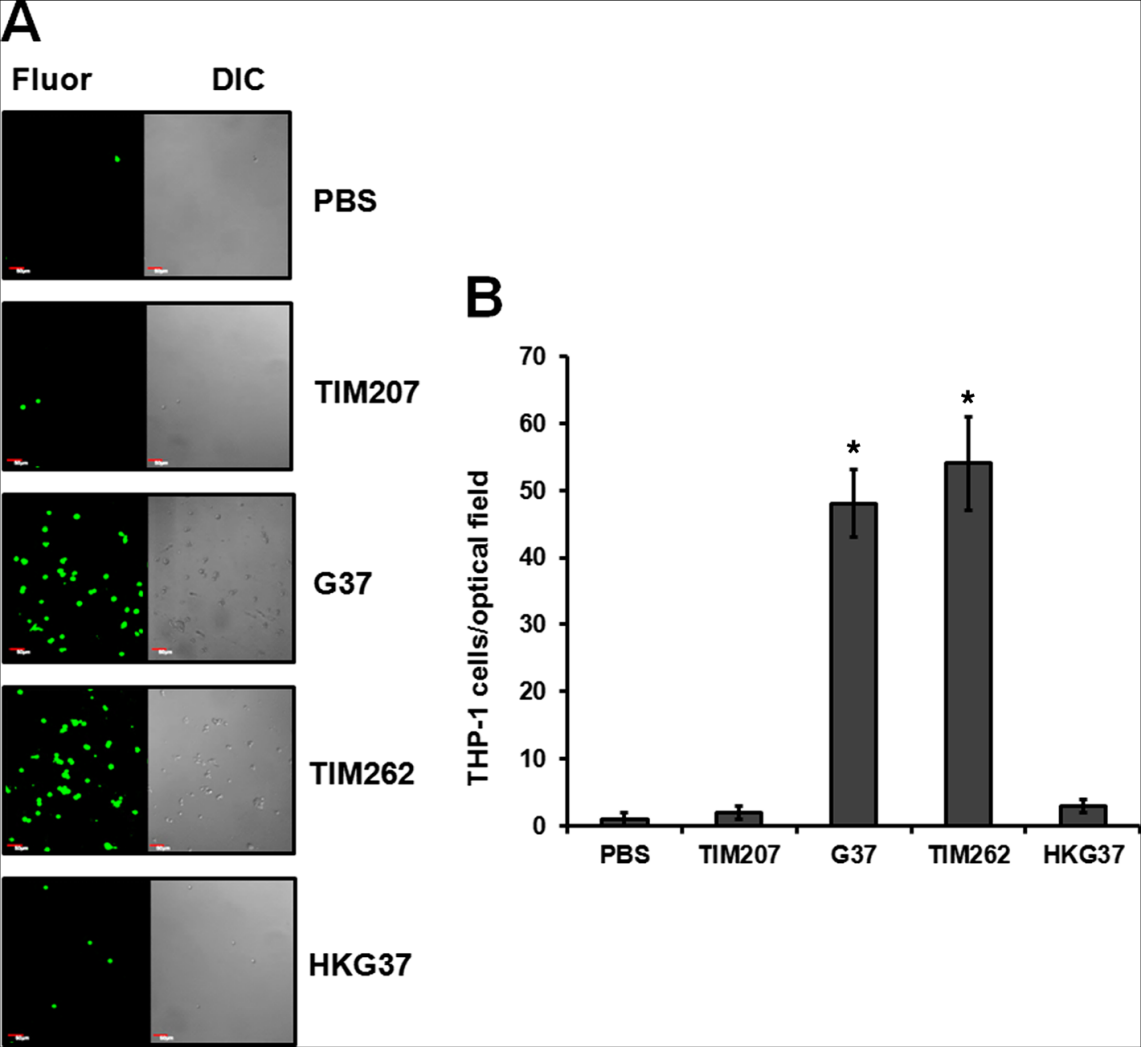
**Differentiation of THP-1 cells by *M. genitalium *strains. A**. Adherent THP-1 cells showing fluorescence. Images of adherent cells were acquired using confocal laser scanning microscope with 10X objective and 488 nm laser. G37, TIM207 and TIM262 are wild type, TIM207 mutant and TIM262 control *M. genitalium* strains, respectively. HKG37 represents heat killed bacteria of wild type *M. genitalium*. Flour, Fluorescence; DIC, Differential interference contrast. **B**. Graph showing the amount of adherent cells for each infection. The numbers of labeled cells in each image were counted using the particle plugin of Image J software. Average cell numbers from five different optical fields and from three independent experiments were used for determining the number of adherent mononuclear cells in each infection. * indicate significant difference from G37 (p≤0.01).

THP-1 cell line is an undifferentiated monocyte cell line from human and its differentiation into macrophages requires incubation with 100 nM of Phorbol-12-myristate-13-acetate (PMA) for 48 to 72 h. Usually, differentiated THP-1 cells adhere to the surface of culture flask, while undifferentiated THP-1 cells only float in the culture medium. We have recently discovered that *M. genitalium* can induce the differentiation of monocytic THP-1 cells into macrophages, similar to that of PMA, and this ability of *M. genitalium* may be affected by the absence of protein like MsrA [54]. To test whether absence of MG207 had any effect on the differentiation of THP-1 cells by *M. genitalium*, we labeled THP-1 cells with CFSE and infected with TIM207 strain and control strains of *M. genitalium*. Figure 7A shows confocal microscopy observation of THP-1 cells adhered to surface of the culture slides due to differentiation induced by *M. genitalium* strains. Although THP-1 cells infected with G37 and TIM262 exhibited higher number of adhered cells, relatively less number of cells adhered with THP-1 cells infected with TIM207 and heat killed bacteria of G37 strain (Figure 7B). This suggested that the product of *MG_207* plays an important role in the induction of differentiation of THP-1 cells by *M. genitalium*.

We presume that phosphorylation of some proteins associated with the differentiation of THP-1 cells is severely affected in this mutant which leads to reduced differentiation of THP-1 cells as compared to wild type. It is unknown at present whether or not the surface proteins like pyruvate dehydrogenase E1 α chain and MG328, which showed altered phosphorylation in this study, have any role in this process but such a possibility does exist. Nevertheless, since differentiation of monocytes is related to modulation of immune responses, the reduced ability of TIM207 strain to differentiate these cells may suggest that this mutant will have only limited ability to alter the immune system to its favor. This hypothesis is supported by the fact that an *msrA* mutant (Δ*MG_408*) of *M. genitalium*, which differentiates THP-1 cells only moderately, could induce only limited amounts of proinflammatory cytokines IL-1β and TNF-α as compared to wild type *M. genitalium* that has the full ability to differentiate THP-1 cells [54]. It is our future goal to investigate whether absence of MG207 protein in *M. genitalium* has any relationship with induction of immune response in the host cells

## Conclusions

In this study, we have shown that the product encoded by *MG_207* in *M. genitalium* is a phosphatase and its absence may affect the phosphorylation of some proteins. We have also provided evidence that absence of MG207 leads to reduced virulence of this bacterium by affecting its ability to cause cytotoxicity and to differentiate monocytic cells. However, the partial adherence phenotype to culture flasks that we observed with TIM207 appears to be significant and what causes this transient phenotype remains a question. Similarly, the factors that led TIM207 to cause reduced cytotoxicity and reduced induction of differentiation of THP-1 cells also remain indefinable at this point. Whether the differentially phosphorylated proteins like MG274, MG328 and MG281 play any role in these processes needs additional investigation.

## Methods

### Bacterial strains and their culture

*Escherichia coli* strains were cultured in LB broth at 37°C with ampicillin 100 μg/ml.

*M. genitalium* wild type strain (G37) was grown in 100 ml of SP-4 medium at 37°C for 72 h in 150 cm^2^ tissue culture flasks (Corning, NY). *M. genitalium* transposon mutant strains TIM207 and TIM262, (kindly provided by Dr. John Glass, J. Craig Venter Institute, Rockville, MD) were also grown similarly in SP-4 medium with 4 μg/ml tetracycline or 50 μg/ml gentamicin.

Adherent *M. genitalium* from culture flasks was washed three times with PBS (pH 7.2) and scraped with cell scrapers (39 cm handle/3 cm blade; Corning, NY). The suspension was centrifuged at 20,000xg for 20 min at 4°C in Sorvall RC 5B centrifuge. The pellets were resuspended in PBS and passed through 18G needles and then through 23G needles to disperse bacterial clumps. The suspensions, diluted to OD_600_=1.0 (which is equivalent to 1 X10^7^ CFU/ml) with PBS, were used to infect cell lines with different multiplicity of infection (MOI).

### Cell lines and their culture

Human cell lines THP-1 (TIB-202) and HeLa (CCL-2) were purchased from American Type Culture Collection (ATCC, Manassas, VA). THP-1 and HeLa cells were cultured in RPMI and Dulbecco’s modified Eagle’s medium (DMEM), respectively, with 10% FBS at 37°C in a humid chamber with 5% CO_2_.

### DNA manipulations

Plasmids from *E. coli* were isolated using QIAprep Spin kit (Qiagen). Genomic DNA from mycoplasma was isolated using DNA isolation kit (Invitrogen). Primers for amplification of *MG_207* gene and subsequent site directed mutagenesis were synthesized at the DNA core facility, The University of Texas Health Science Center at San Antonio (UTHSCSA). The whole gene encoding MG207 was amplified by PCR using primers MG_207EX1 (5´-ACGCATATGCAAAACAAACTGATTAAGGTT-3´) and MG_207EX2 (5´-CAGTCGGATCCGTTAACTAACTTTTGAAGCTTG-3´) and *M. genitalium* genomic DNA as template. This fragment was cloned into pCR 2.1 to result in pMG207. The gene *MG_207* has a TGA codon for tryptophan residue, which will be recognized as stop codon by *E. coli*, and this needed modification into TGG to express the gene in *E. coli*. To do this modification (point mutation), we used QuikChange Site-Directed Mutagenesis Kit (Stratagene) and primers MG_207M1 (5´-CAAAATGCTACTTTTTGGGTGGCAGGTAACAAC-3´) and MG_207M2 (5´-GTTGTTACCTGCCACCCAAAAAGTAGCATTTTG-3´). Plasmid pMG207 served as the template for point mutation. Subsequent to point mutation, the newly synthesized plasmid DNA (pMG207A) was transformed into *E. coli*, plasmid isolated and the sequence of the insert region was verified to confirm the point mutation. The coding region of *MG_207* from pMG207A was digested with NdeI and BamHI and the fragment cloned into similarly cut pET16b expression vector. This plasmid (pMG207EX) was transformed into *E. coli* BL21 (DE3) strain to overexpress His_10_MG207 protein.

### Southern hybridization

To reconfirm the insertion of transposon Tn4001 in *MG_207*, we performed Southern hybridization. Briefly, chromosomal DNA from *M. genitalium* G37 and TIM207 was cut with SpeI and separated in 1% agarose gels. The separated DNA fragments were transferred to Zeta probe membranes (Bio-Rad) by Southern blotting and crosslinked with UV. Prehybridization of the membranes was performed in a solution containing 50% formamide, 0.12 M Na_2_HPO_4_, 0.25 M NaCl, and 7% (wt/vol) sodium dodecyl sulfate (SDS) for 4 h. Hybridization of the membranes was done in the same solution with [α-^32^P]dCTP labeled probe DNA of *MG_207* or *gentamicin* gene for overnight at 42°C. The membranes were washed at 42°C (each wash for 15 min with solutions A (2X SSC with 0.1% SDS), B (0.5X SSC with 0.1% SDS) and C (0.1X SSC with 0.1% SDS) for three times. Afterwards, the membranes were exposed to X-ray films for autoradiography.

### Overexpression of MG207 in *E. coli*

Overexpression and purification of recombinant MG207 protein using pET16b were performed as detailed before [55,56]. Briefly, *E. coli* strain BL21 (DE3) harboring the pMG207EX was induced with 0.5 mM IPTG at 37°C to overexpress the protein. The overexpressed protein was purified with Ni-NTA affinity column chromatography (Qiagen). The *E. coli* extracts and purified protein were separated on 12% SDS-PAGE to assess the expression and purification. The purified recombinant protein was designated as His_10_MG207. All purification and desalting procedures were performed with buffers based on Tris–HCl pH8.0 and use of phosphate buffer was avoided.

### Enzyme assays

To determine if the overexpressed and purified His_10_MG207 was functional, we performed phosphatase assay with *p*-nitrophenyl phosphate (pNPP) as substrate (Sigma-Aldrich, St. Louis, MO). The assay was conducted in 96 well plates and the assay mixture (120 μl) contained 1 mM pNPP in 20 mM Tris–HCl pH 8.0, 5 mM MgCl_2_ and His_10_MG207 protein. Control reactions had no protein or heat inactivated His_10_MG207. Each reaction was done in triplicate wells. The reaction mixtures were incubated at 37°C for 1 h and the yellow color, developed due to the hydrolysis of pNPP, was read at 405 nm using a Spectramax plate reader (Molecular Devices, Sunnyvale, CA).

To determine the specificity of His_10_MG207 towards serine or threonine residue, we used Alkaline/Acid Phosphatase assay kit (Millipore, Temecula, CA). This uses synthetic peptides for serine phosphate (RRApSSVA) and threonine phosphate (KRpTIRR) as substrates for the enzyme assay. The reactions were done as described by the manufacturer in 96 well plates, except that the reaction mixture had MgCl_2_ instead of NiCl_2_. Amount of phosphate released was calculated using phosphate reference standards supplied with the kit.

### SDS-PAGE and immunoblot

Premade SDS-PAGE gels (NuPAGE 12% Bis-Tris gel, Invitrogen, Carlsbad, CA) were used to separate proteins from *E. coli* and *M. genitalium* for coomassie staining of proteins and for Western blot. In these gels 50 μg of total protein was loaded per well. Protein concentration was determined by BCA method (Pierce). Western blots were probed with anti-MG207 rabbit antiserum (1:500 dilutions) to detect MG207 protein of *M. genitalium* strains. This rabbit antiserum was generated against purified His_10_MG207 protein using a commercial source (Alpha Diagnostic International Inc., San Antonio).

### Two-dimensional gel analysis of proteins

Two-dimensional (2-D) gel analyses of total proteins of *M. genitalium* G37 and TIM207 strains were performed by Kendrick Lab Inc., (Madison, WI). Fifty μg of total proteins were separated by isoelectric focusing [IEF] in glass tubes with an inner diameter of 2.0 mm. The IEF gel contained 2% pH 4–6 ampholines (Servalytes, Serva, Heidelberg, Germany) and 2% pH 5–8 ampholines (GE Healthcare). After IEF, gels were equilibrated for 10 min in buffer “0” (10% glycerol, 50 mm dithiothreitol, 2.3% SDS and 0.0625 M Tris, pH 6.8). Thereafter, each tube gel was sealed to the top of a stacking gel that was overlaid above 10% SDS-PAGE acrylamide gels (slab gels, 0.75 mm thick) and gels were run for about 4 h at 15 mA/gel. The gels were then fixed twice in 50% methanol 10% acetic acid solution and stained with Pro-Q Diamond for phosphoproteins. Images of the gels were acquired by scanning the gels with Bio-Rad Molecular Imager FX ProPlus scanner. After destaining, the gels were stained with Sypro Ruby (Molecular Probes) and again scanned with Bio-Rad Molecular Imager FX ProPlus scanner to obtain the images of total proteins. The following proteins (Sigma Chemical Co., St. Louis, MO) were used as molecular weight standards: myosin (22,000), phosphorylase A (94,000), catalase (60,000), actin (43,000), carbonic anhydrase (29,000) and lysozyme (14,000).

### Mass spectrometry

Mass spectrometry analyses were conducted in our core facility at UTHSCA. Pro-Q Diamond-stained gel spots were manually excised and digested *in situ* with trypsin (Promega, modified) in 40 mM NH4HCO3 overnight at 37°C. The digests were analyzed by capillary HPLC-electrospray ionization tandem mass spectrometry (HPLC-ESI-MS/MS) using a Thermo Fisher LTQ linear ion trap mass spectrometer fitted with a New Objective PicoView 550 nanospray interface. On-line HPLC separation was accomplished with an Eksigent NanoLC micro HPLC: column, PicoFrit™ (New Objective; 75 μm i.d.) packed to 11 cm with Vydac 218MSB5 (5 μm, 300 Å) using a scan strategy in which a survey scan was acquired followed by data-dependent collision-induced dissociation (CID) of the seven most intense ions in the survey scan above a set threshold. The uninterpreted CID spectra were searched by means of Mascot (Matrix Science) against the Swiss-Prot database [2011_03 (525,997 sequences; 185,874,894 residues)] as follows: enzyme, trypsin, one missed cleavage allowed; precursor and fragment ion mass tolerances, ± 1.5 Da and ± 0.8 Da, respectively; variable modifications, methionine oxidation and phosphorylation of serine, threonine and tyrosine. Cross correlation of the Mascot results with X! Tandem and determination of probabilities for peptide assignments and protein identities were accomplished by Scaffold™ (Proteome Software).

**Attachment of mycoplasmas to the HeLa cells:** HeLa cells (2.5 × 10^5^) were grown on square cover slides in 6 well tissue culture plates (Corning, NY). *M. genitalium* strains were labeled with Fluorescein isothiocyanate isomer I (FITC: Sigma-Aldrich, St. Louis, MO) as described before [54] and infected with an MOI of 1:25 for 1 h at 37°C. The cell monolayer was then washed three times with PBS and images captured using at 488 nm in an inverted laser microscope (Olympus FV1000) with 20 X objective (NA 0.75).

### Cytotoxic assay

Cytotoxicity of *M. genitalium* strains was assessed by infecting HeLa cell line as reported earlier [54]. Briefly, HeLa cells (2.5 × 10^5^) were grown on coverslips in 6 well plates for 24 h and then infected with wild type G37 and mutant (TIM207 and TIM262) *M. genitalium* strains with MOI=50 for 2–3 h. Heat killed *M. genitalium* (HKG37) was used as control. Cytotoxic effect was determined by evaluating the integrity of the infected cells using differential interference contrast [57] at 488 nm in an inverted laser scanning confocal microscope (Olympus FV1000) with 20X objective.

### Determination of H_2_O_2_ in *M. genitalium* strains

Production of H_2_O_2_ by mycoplasma strains was measured by colorimetric ferrous ion oxidation in xylenol orange [FOX] method [58,59]. Protein samples from strains of *M. genitalium* were used as the source for H_2_O_2._ Protein content of samples was determined using Pierce BCA Protein Assay Kit (Pierce). Equal amount of protein samples (each 25 μl) and cold FOX reagent (250 μl) were mixed and incubated for 30 min at room temperature. After incubation, absorbance was measured at 560 nm. The amount of hydrogen peroxide in each sample was determined using a standard curve generated with known amounts of H_2_O_2._ The results were expressed as μmoles H_2_O_2_/per μg protein.

### Differentiation of monocytic THP-1 cells by *M. genitalium* strains

THP-1 cells were labeled with carboxyfluorescein diacetate succinimidyl ester (CFSE) and cells (0.5X10^5^) were plated on 4 chamber 1.5 German cover glass slides (Nunc, Rochester, NY). The cells were then infected with (MOI 1:5) *M. genitalium* (G37 or TIM207 or TIM262 or HKG37) for 1 h. After incubation, the chambers were washed with PBS to remove non-adherent cells. Cells adhering to the cover slips were examined under FV1000 laser scanning inverted confocal microscope (Olympus, Japan) with 20X objective. Images were acquired and labeled cells in each image was counted using the NIH analyze particle plug-in of Image J software.

### Statistical analysis

The data were analyzed by paired *t-*test using graphpad prism software.

## Competing interests

The authors have no competing interests to declare.

## Authors’ contributions

SD designed the study; MAM performed the overexpression of MG207 and phosphatase assay; KD performed all experiments involving microscopes, *M. genitalium* viability assays and glycerol utilization assays; SS performed the Southern blot and FOX assay, LAM helped in designing some experiments and writing the manuscript; KD analyzed the data and created the figures; SD wrote the manuscript. All authors have read and approved the manuscript.

## Supplementary Material

Additional file 1: Figure 1 Viability of *M. genitalium* strains based on color change assay. *M. genitalium* G37, TIM207 and TIM262 were grown and harvested as described in method section. The bacteria were resuspended in appropriate amount of PBS to give an OD600 =1.0. Different volume of the inoculum, as indicated in the x-axis were added to 200 μl of SP-4 medium in a 96 well plate and incubated at 37°C for 6 h. Color change of SP-4 medium, due to the growth of mycoplasma, from red to orange was monitored by reading the plate at 620 nm in a microplate reader. Solid grey bars, dotted bars, solid black bars and horizontal stripped bars indicate absorbance (A620) of PBS, TIM207, G37 and TIM 262 respectively. The results indicate that there is no significant difference in viability between the strains at the time of harvest. Click here for file

Additional file 2: Table S1 Mass spectrometry of analysis of 2D spots. Click here for file

Additional file 3: Figure S2 Growth of *M. genitalium* G37 and TIM207 strains in the presence of glucose and glycerol. G37 and TIM207 was grown in a T-25 flask with SP-4 medium with either 1% (v/v) glucose or glycerol as carbon source until the color of the medium turns yellow (approximately 5 days, four different flasks for each strains). The bacteria were collected by scrapping and by centrifugation at 12,000 rpm for 15 min. The cells were washed two times in sterile PBS and finally suspended thoroughly with 23G syringe in 1 ml of sterile PBS and OD at 600 nm recorded. The solid bars and stripped bars indicate absorbance (A600) of either of strains grown in glucose and glycerol, respectively. “*” = p≤ 0.05 between TIM207 grown in glucose vs glycerol. Click here for file
