# Supplementary material for: A serine/threonine phosphatase encoded by MG_207 of Mycoplasma genitalium is critical for its virulence
Source: BMC Microbiol. 2013 Feb 21;13:44. doi: 10.1186/1471-2180-13-44 (PMC3639085; doi:10.1186/1471-2180-13-44)
Supplement: Additional file 1: Figure 1 — Viability of M. genitalium strains based on color change assay. M. genitalium G37, TIM207 and TIM262 were grown and harvested as described in method section. The bacteria were resuspended in appropriate amount of PBS to give an OD600 =1.0. Different volume of the inoculum, as indicated in the x-axis were added to 200 μl of SP-4 medium in a 96 well plate and incubated at 37°C for 6 h. Color change of SP-4 medium, due to the growth of mycoplasma, from red to orange was monitored by reading the plate at 620 nm in a microplate reader. Solid grey bars, dotted bars, solid black bars and horizontal stripped bars indicate absorbance (A620) of PBS, TIM207, G37 and TIM 262 respectively. The results indicate that there is no significant difference in viability between the strains at the time of harvest. [file 1471-2180-13-44-S1.pptx]

## Slide 1
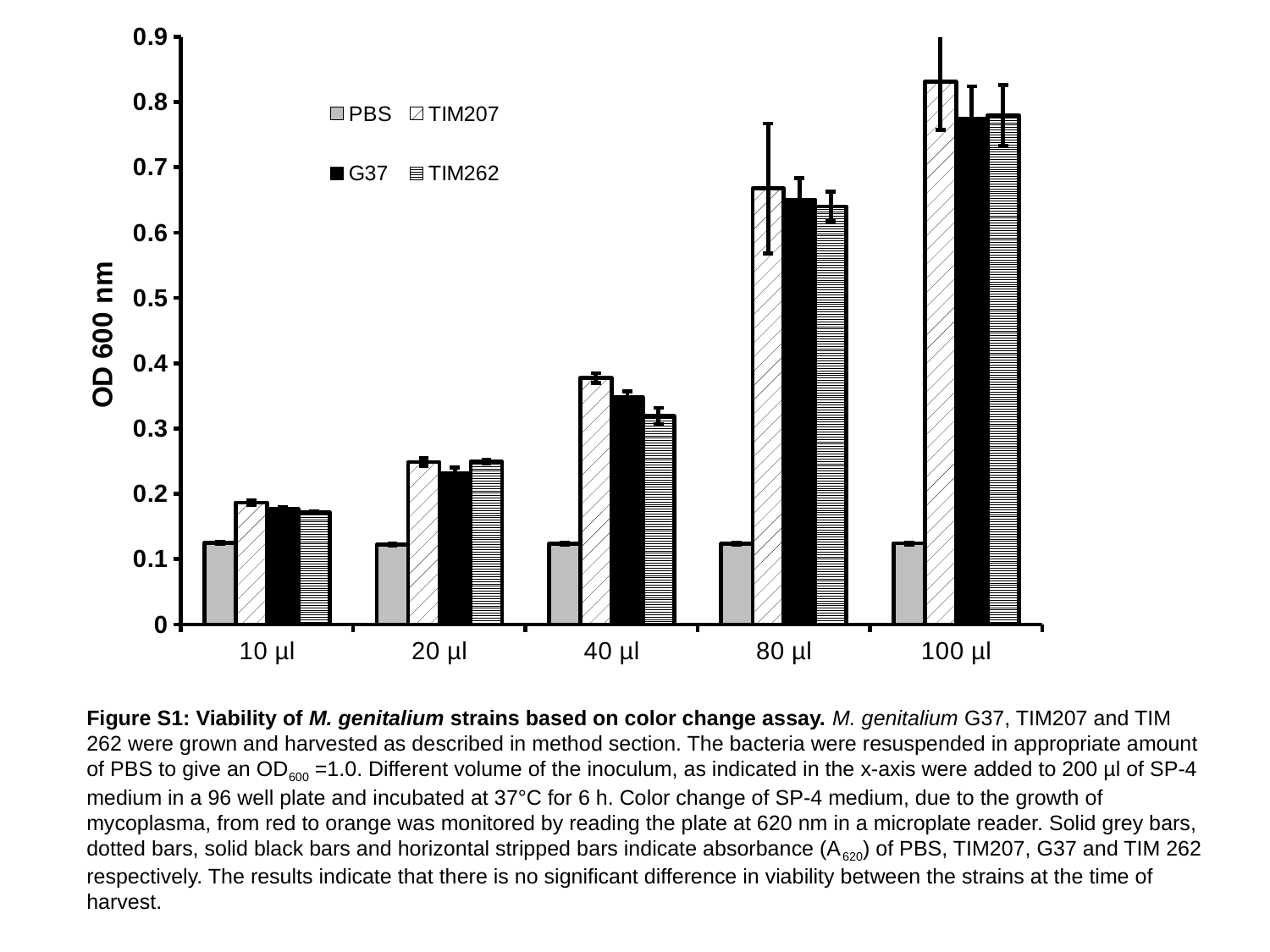

### Chart
| Category | PBS | TIM207 | G37 | TIM262 |
|---|---|---|---|---|
| 10 µl | 0.12502 | 0.18657 | 0.17694 | 0.17191 |
| 20 µl | 0.12211 | 0.24857 | 0.23114 | 0.24961 |
| 40 µl | 0.12333 | 0.37725 | 0.34751 | 0.31892 |
| 80 µl | 0.12358 | 0.66773 | 0.65032 | 0.64 |
| 100 µl | 0.12398 | 0.83109 | 0.77388 | 0.77981 |Figure S1: Viability of M. genitalium strains based on color change assay. M. genitalium G37, TIM207 and TIM 262 were grown and harvested as described in method section. The bacteria were resuspended in appropriate amount of PBS to give an OD600 =1.0. Different volume of the inoculum, as indicated in the x-axis were added to 200 µl of SP-4 medium in a 96 well plate and incubated at 37°C for 6 h. Color change of SP-4 medium, due to the growth of mycoplasma, from red to orange was monitored by reading the plate at 620 nm in a microplate reader. Solid grey bars, dotted bars, solid black bars and horizontal stripped bars indicate absorbance (A620) of PBS, TIM207, G37 and TIM 262 respectively. The results indicate that there is no significant difference in viability between the strains at the time of harvest.
