# Supplementary material for: A serine/threonine phosphatase encoded by MG_207 of Mycoplasma genitalium is critical for its virulence
Source: BMC Microbiol. 2013 Feb 21;13:44. doi: 10.1186/1471-2180-13-44 (PMC3639085; doi:10.1186/1471-2180-13-44)
Supplement: Additional file 2: Table S1 — Mass spectrometry of analysis of 2D spots. [file 1471-2180-13-44-S2.docx]

| **S.No** | **Identified Proteins** | **Accession Number** | **Molecular Weight** | **Peptides for each Spot**  **1 2 3 4 5** | | | | |
| --- | --- | --- | --- | --- | --- | --- | --- | --- |
| 1 | Uncharacterized protein MG328 | Y328_MYCGE | 88 kDa | 0 | 0 | 103 | 3 | 0 |
| 2 | Pyruvate dehydrogenase E1 component subunit alpha | ODPA_MYCGE | 41 kDa | 10 | 66 | 0 | 0 | 0 |
| 3 | Uncharacterized protein MG281 | Y281_MYCGE | 62 kDa | 6 | 12 | 0 | 34 | 8 |
| 4 | Trypsin | TRYP_PIG | 24 kDa | 6 | 8 | 5 | 0 | 5 |
| 5 | Putative type-1 restriction enzyme specificity protein MG438 | T1SX_MYCGE | 44 kDa | 0 | 0 | 0 | 9 | 8 |
| 6 | Thymidine phosphorylase | TYPH_MYCGE | 46 kDa | 0 | 0 | 0 | 6 | 11 |
| 7 | 60 kDa chaperonin | CH60_MYCGE | 58 kDa | 0 | 16 | 0 | 0 | 0 |
| 8 | Oligopeptide transport ATP-binding protein OppD | OPPD_MYCGE | 45 kDa | 0 | 0 | 0 | 2 | 4 |
| 9 | Phosphoglycerate kinase | PGK_MYCGE | 45 kDa | 0 | 0 | 0 | 2 | 4 |
| 10 | Uncharacterized lipoprotein MG412 | Y412_MYCGE | 42 kDa | 0 | 4 | 0 | 0 | 0 |
| 11 | Uncharacterized protein MG075 | Y075_MYCGE | 116 kDa | 0 | 2 | 0 | 0 | 0 |
| 12 | Ribose-phosphate pyrophosphokinase | KPRS_MYCGE | 39 kDa | 0 | 4 | 0 | 0 | 0 |
| 13 | Dihydrolipoyllysine-residue acetyltransferase component of pyruvate dehydrogenase complex pdhC | ODP2_MYCGE | 41 kDa | 0 | 3 | 0 | 0 | 0 |
| 14 | Serine hydroxymethyltransferase | GLYA_MYCGE | 45 kDa | 0 | 0 | 0 | 0 | 3 |
| 15 | Tyrosyl-tRNA synthetase | SYY_MYCGE | 46 kDa | 0 | 3 | 0 | 0 | 0 |
| 16 | Ribonucleoside-diphosphate reductase subunit beta | RIR2_MYCGE | 39 kDa | 0 | 2 | 0 | 0 | 0 |
| 17 | Elongation factor G | EFG_MYCGE | 77 kDa | 0 | 2 | 0 | 0 | 0 |
| 18 | Pyruvate dehydrogenase E1 component subunit beta | ODPB_MYCGE | 36 kDa | 0 | 2 | 0 | 0 | 0 |

**Table S1. Mass spectrometry of analysis of 2D spots**
