# Supplementary material for: A serine/threonine phosphatase encoded by MG_207 of Mycoplasma genitalium is critical for its virulence
Source: BMC Microbiol. 2013 Feb 21;13:44. doi: 10.1186/1471-2180-13-44 (PMC3639085; doi:10.1186/1471-2180-13-44)
Supplement: Additional file 3: Figure S2 — Growth of M. genitalium G37 and TIM207 strains in the presence of glucose and glycerol. G37 and TIM207 was grown in a T-25 flask with SP-4 medium with either 1% (v/v) glucose or glycerol as carbon source until the color of the medium turns yellow (approximately 5 days, four different flasks for each strains). The bacteria were collected by scrapping and by centrifugation at 12,000 rpm for 15 min. The cells were washed two times in sterile PBS and finally suspended thoroughly with 23G syringe in 1 ml of sterile PBS and OD at 600 nm recorded. The solid bars and stripped bars indicate absorbance (A600) of either of strains grown in glucose and glycerol, respectively. “*” = p≤ 0.05 between TIM207 grown in glucose vs glycerol. [file 1471-2180-13-44-S3.pptx]

## Slide 1
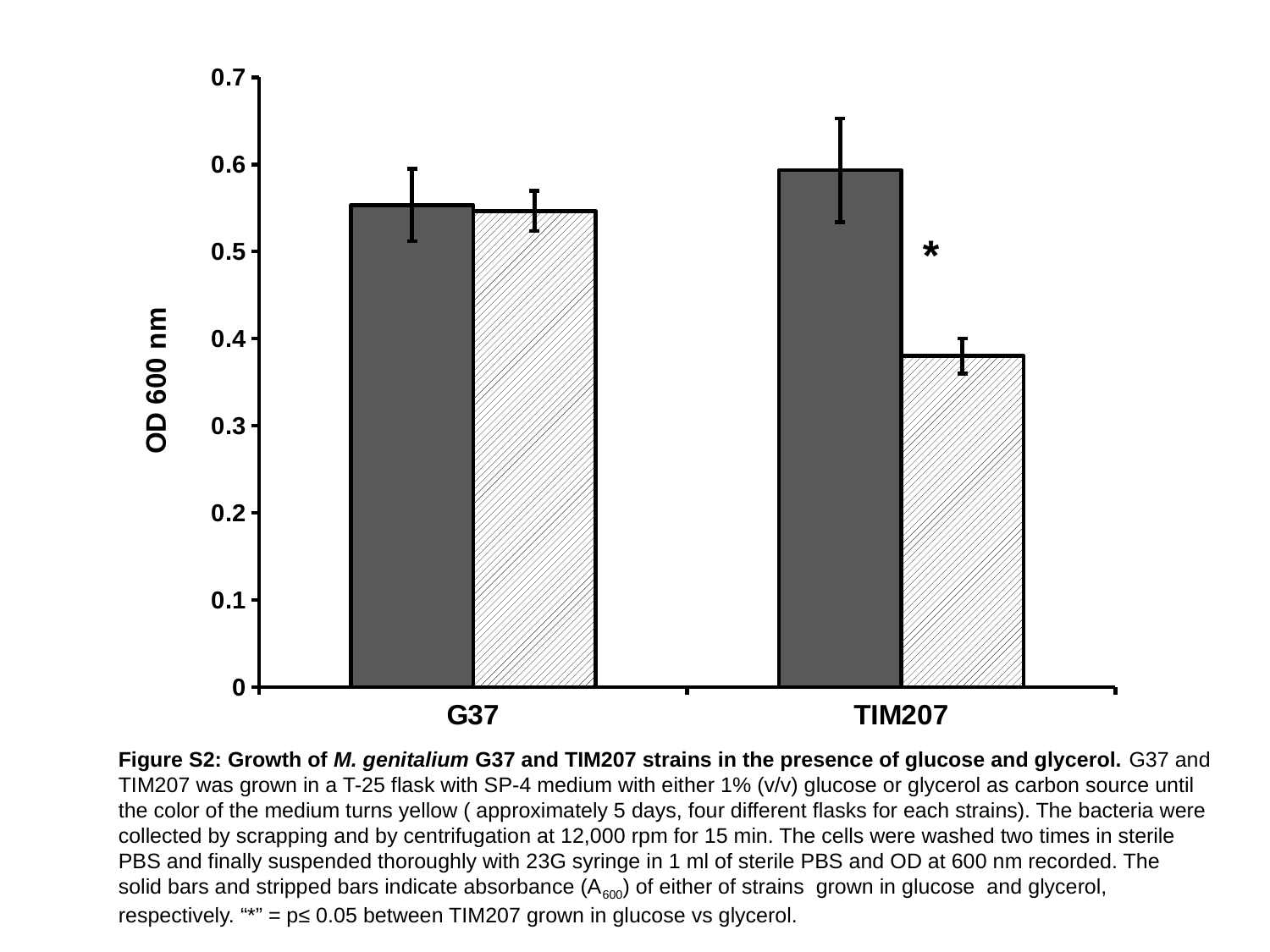

### Chart
| Category | Glucose | Glycerol |
|---|---|---|
| G37 | 0.5533 | 0.5466 |
| TIM207 | 0.5933 | 0.38 |*
Figure S2: Growth of M. genitalium G37 and TIM207 strains in the presence of glucose and glycerol. G37 and TIM207 was grown in a T-25 flask with SP-4 medium with either 1% (v/v) glucose or glycerol as carbon source until the color of the medium turns yellow ( approximately 5 days, four different flasks for each strains). The bacteria were collected by scrapping and by centrifugation at 12,000 rpm for 15 min. The cells were washed two times in sterile PBS and finally suspended thoroughly with 23G syringe in 1 ml of sterile PBS and OD at 600 nm recorded. The solid bars and stripped bars indicate absorbance (A600) of either of strains grown in glucose and glycerol, respectively. “*” = p≤ 0.05 between TIM207 grown in glucose vs glycerol.
